# A review of *Brucella *infection in marine mammals, with special emphasis on *Brucella pinnipedialis *in the hooded seal (*Cystophora cristata*)

**DOI:** 10.1186/1297-9716-42-93

**Published:** 2011-08-05

**Authors:** Ingebjørg H Nymo, Morten Tryland, Jacques Godfroid

**Affiliations:** 1 Department of Food Safety and Infection Biology, Section of Arctic Veterinary Medicine, the Norwegian School of Veterinary Science, Stakkevollveien 23, N-9010 Tromsø, Norway; 2 Member of Fram - High North Research Centre for Climate and the Environment, Hjalmar Johansens gate 14, N-9296 Tromsø, Norway

## Abstract

*Brucella *spp. were isolated from marine mammals for the first time in 1994. Two novel species were later included in the genus; *Brucella ceti *and *Brucella pinnipedialis*, with cetaceans and seals as their preferred hosts, respectively. *Brucella *spp. have since been isolated from a variety of marine mammals. Pathological changes, including lesions of the reproductive organs and associated abortions, have only been registered in cetaceans. The zoonotic potential differs among the marine mammal *Brucella *strains. Many techniques, both classical typing and molecular microbiology, have been utilised for characterisation of the marine mammal *Brucella *spp. and the change from the band-based approaches to the sequence-based approaches has greatly increased our knowledge about these strains. Several clusters have been identified within the *B. ceti *and *B. pinnipedialis *species, and multiple studies have shown that the hooded seal isolates differ from other pinniped isolates. We describe how different molecular methods have contributed to species identification and differentiation of *B. ceti *and *B. pinnipedialis*, with special emphasis on the hooded seal isolates. We further discuss the potential role of *B. pinnipedialis *for the declining Northwest Atlantic hooded seal population.

## Table of contents

1. Genus *Brucella *- hosts and reservoirs 4

2. Infection with *B. ceti *and *B. pinnipedialis *6

2.1. Pathology in association with *B. ceti *infection in cetaceans 7

2.1.1. Gross pathology 7

2.1.2. Pathology in reproductive organs 8

2.2. Experimental infections in livestock and laboratory animals with marine mammal strains of *Brucella *8

2.3. The zoonotic potential of marine mammal strains of *Brucella *9

3. Characterization of *B. ceti *and *B. pinnipedialis *10

3.1. Classical typing approaches 11

3.2. Identification of *B. ceti *and *B. pinnipedialis *as part of the *Brucella *genus 11

3.3. Molecular characterisation: band-based approaches 12

3.3.1. Pulsed-Field Gel Electrophoresis (PFGE) and early Polymerase Chain Reaction (PCR) based approaches 12

3.3.2. Studies of the outer membrane protein genes (*omp*-genes) 13

3.3.3. Studies of insertion sequence 711 (*IS*711) 14

3.4. Molecular characterisation: sequence-based approaches 15

3.4.1. Analysis based on tandem repeats 16

3.4.2. Multi locus sequence analysis (MLSA) 17

3.4.3. Single-Nucleotide Polymorphism (SNP) typing 18

3.4.4. Whole genome comparison 18

4. *B. pinnipedialis *in hooded seals 20

4.1. The biology of the hooded seal 20

4.2. Infection in hooded seals with *B. pinnipedialis *21

4.3. Characterisation of the hooded seal strains of *B. pinnipedialis *22

5. Conclusions and future perspectives 22

## 1. Genus *Brucella *- hosts and reservoirs

The Genus *Brucella*, belonging to the class α-proteobacteria and order *Rhizobiales *[1,2], contains gram-negative, non-motile, facultative intracellular, small coccobacilliary bacteria. Classically there were six species; *Brucella melitensis*, *B. abortus*, *B. suis*, *B. ovis*, *B. neotomae *and *B. canis *[3]. Due to the high homogeneity demonstrated by DNA-DNA hybridisation studies, it was suggested that the entire genus should be one species [4]. This was accepted by the Subcommittee on the Taxonomy of *Brucella *in 1986 [5], but not by the *Brucella *research community. In 2003, the Subcommittee on the Taxonomy of *Brucella *again accepted the six classical species [6]. Recently, four additional species were added to the genus, *Brucella ceti *and *Brucella pinnipedialis*, with cetaceans and seals as their preferred hosts, respectively [7], *Brucella microti*, isolated from the common vole (*Microtus arvalis*) in the Czech Republic [8], from soil in the same area years later [9], and from mandibular lymph nodes of wild red foxes (*Vulpes vulpes*) in Austria [10] and *Brucella inopinata*, isolated from a breast implant wound of a woman with clinical signs of brucellosis [11]. A prospective *Brucella *species has been isolated from native rat species in Australia, but has not yet been included in the genus [12].

Transmission of *Brucella *spp. between animals usually takes place through contact with aborted, infected material, most often through ingestion, but also through respiratory exposure, conjunctival inoculation and infection through damaged skin or mucosal membranes. *Brucella *spp. can also be transmitted during breeding and lactation [13,14]. *Brucella *spp. do not multiply outside the host, but persist for years in frozen aborted foetuses or placentas, for months in moist conditions at 10-15°C, and for hours at 45-50°C [15]. An exception is *B. microti*, which have a long-term reservoir in soil [9].

Acute infection with *Brucella *spp. is initiated by the entrance of the bacteria into the bloodstream after which they are engulfed by circulating polymorphonuclear cells and macrophages, evading the bactericidal systems of the cells. Due to the bacterium's predilection for cells producing erythritol, *Brucella *spp. localise in the pregnant uterus of ruminants, inducing abortions in late pregnancy and premature births [3,13,14]. After the initial acute phase, brucellosis in the primary host usually becomes latent, although abortions in the subsequent gestation may happen. Females may shed the bacterium periodically through milk and uterine and vaginal discharges in subsequent parturitions, as described in ewes [16]. In males, epididymitis and orchitis are typical clinical signs [3,13,14].

*Brucella *spp. belonging to the six classical species have been isolated from a variety of terrestrial wildlife species, including bison (*Bison bison*), muskoxen (*Ovibos moschatus*), elk/wapiti (*Cervus elaphus canadensis*), moose (*Alces alces*), reindeer (*Rangifer tarandus tarandus*), barren ground caribou (*Rangifer tarandus groenlandicus*), white tailed deer (*Odocoileus virginianus*), roe deer (*Capreolus capreolus*), chamois (*Rupicapra rupicapra*), African buffalo (*Syncerus caffer*), impala (*Aepyceros melampus*), waterbuck (*Kobus elipsiprymnus*), red fox, Pampas fox (*Dusicyon gymnocercus*), Patagonian Gray Fox (*Dusicyon griseus*), racoon (*Procyon lotor*), opossum (*Didelphis virginiana*), European hare (*Lepus europaeus*), feral pig (*Sus scrofa*), wild boar (*Sus scrofa*) and capybara (*Hydrochaeris hydrochaeris*) [17,18]. Nile catfish (*Clarias gariepinus*) have been experimentally infected with *B. melitensis *biovar 3. The fish seroconverted and *Brucella *spp. were isolated from internal organs, but transmission to non-infected sentinel fish did not occur [19]. Recently, the first detection of naturally occurring *Brucella *spp. in fish was reported when *B. melitensis *biovar 3 was cultured from skin swabs from Nile catfish, and PCR confirmed the identity of the bacterium. Antibodies against *Brucella *spp. were also detected in the fish [20].

*Brucella *spp. have zoonotic potential. The main source of human infection is production animals. The most frequently reported cause of zoonotic *Brucella *infection and the most clinically important is *B. melitensis*. *B. suis *(biovar 1, 3 and 4) and *B. abortus *are also zoonotic. In humans, *Brucella *spp. produce varied and nonspecific symptoms, most commonly undulant fever [3,14,21]. Transmission of *Brucella *spp. from wildlife to humans has been reported [17,22]. *B. suis *biovar 4, the agent for brucellosis in reindeer and caribou, has been isolated from clinically ill humans in northern Canada and Alaska, having caribou as part of their diet, and from caribou, reindeer and muskoxen in the same area [23,24]. Characterisation of *Brucella *spp. isolated from Alaskan Eskimos who consumed reindeer meat, showed that this was *B. suis *biovar 4, indicating zoonotic transmission [25]. Zoonotic transmission of *B. canis *is rare and zoonotic transmission of *B. ovis *has not been reported [3]. Both *B. canis *and *B. ovis *have a rough lipopolysaccharide (LPS) phenotype which is associated with reduced pathogenicity compared to bacterial species with the smooth LPS [26,27].

## 2. Infection with *B. ceti *and *B. pinnipedialis*

*Brucella *spp. were initially isolated from marine mammals in 1994, from a harbour seal (*Phoca vitulina*), a harbour porpoise (*Phocoena phocoena*) and a common dolphin *(Delphinus delphis*) in Scotland [28], and an aborted foetus from a captive bottlenose dolphin (*Tursiops truncatus*) in California, USA [29]. Since then, *Brucella *spp. have been isolated from (Additional file 1. Isolation of *Brucella *spp. from marine mammals) and serologically indicated in (Additional file 2. Serological evidence of *Brucella *spp. in marine mammals) a wide range of marine mammal species from most parts of the world. In this review, we summarise and discuss the occurrence of *B. ceti *and *B. pinnipedialis *in marine mammals, the pathology reported in marine mammals and the possible implications of a *B. pinnipedialis *infection for the hooded seal, especially the Northeast Atlantic population. We also discuss the zoonotic potential of *B. ceti *and *B. pinnipedialis*. Further, we describe how different molecular methods have contributed to species identification and differentiation of *B. ceti *and *B.pinnipedialis*, with emphasis on hooded seal isolates, and how these methods can be used to increase our understanding of the marine mammal brucellae.

### 2.1. Pathology in association with *B. ceti *infection in cetaceans

Gross pathology in association with *Brucella *infection in marine mammals is seen exclusively in cetaceans. The infection may have several outcomes and a wide range of pathological changes have been reported (Additional file 1. Isolation of *Brucella *spp. from marine mammals).

#### 2.1.1. Gross pathology

*B. ceti *and *B. pinnipedialis *have been isolated from a variety of organs in apparently healthy marine mammals [30-34]. *B. ceti *has been associated with a range of pathological changes in cetaceans, including blubber abscesses, subcutaneous lesions, skin lesions, hepatic and splenic necrosis and inflammation, macrophage infiltration in the liver and spleen, pneumonia, peritonitis and lymph node inflammation and necrosis. Pathologic changes, including spinal discospondylitis, meningoencephalitis, meningitis, choroiditis, altered cerebrospinal fluid and remodeling of the occipital condyles, often associated with neurologic symptoms, have been reported several times in cetaceans [35-44]. *B. ceti *and *B. pinnipedialis *have been isolated from lungworms found in cetacean and pinniped lungs, respectively [44-48].

#### 2.1.2. Pathology in reproductive organs

*B. ceti *has been isolated from aborted foetuses and reproductive organs in captive bottlenose dolphins [29] with placentitis [49], and from the reproductive organs, milk and foetus of stranded striped dolphins [37]. Bacteria have also been isolated from the uterus and a dead foetus of a stranded striped dolphin with placentitis [50]. Immunohistochemical investigations with polyclonal antiserum and electron microscopy revealed *B. ceti *in a genital ulcer, uterus, mammary gland and milk from a stranded harbour porpoise with endometritis and signs of a recent pregnancy [44]. *B. ceti *has also been isolated in association with mastitis and endometritis in cetaceans [35]. Suppurative granulomatous lesions have been found in both female and male reproductive organs in seropositive baleen whales [51]. *B. ceti *and *B. pinnipedialis *have also been isolated from the testes [41], the uterus [40] and the mammary gland [42] of cetaceans and pinnipeds without any apparent pathology.

### 2.2. Experimental infections in livestock and laboratory animals with marine mammal strains of *Brucella*

Abortion has been experimentally induced in cattle after intravenous injection of *B. pinnipedialis *isolated from a Pacific harbour seal [52]. Ten piglets were challenged with marine mammal *Brucella *spp. [53], originally isolated from a human [54]. No pathological changes were detected, transient and low antibody titres were recorded from three of the piglets, and bacteria were isolated from one lymph node of three other piglets. The marine mammal isolate was unable to establish a sustained infection in the piglets [53]. Experimental infection in pregnant sheep with marine mammal *Brucella *isolates from seal, porpoise and dolphin resulted in limited seroconversion in the sheep. The bacteria were not able to fully establish an infection in the sheep and showed limited pathogenicity [55]. Infection of guinea pigs with the same three isolates resulted in splenomegaly and antibody titres comparable to the *B. abortus *reference strains [55]. Another guinea pig study indicated that *B. ceti *isolated from the aborted foetus of a bottlenose dolphin was less virulent than the reference strains *B. abortus *and *B. melitensis *[29]. No studies have till now been reported in the experimental mouse model of infection.

### 2.3. The zoonotic potential of marine mammal strains of *Brucella*

There are indications that certain of the marine mammal *Brucella *spp. have zoonotic potential. A laboratory worker cultivating marine mammal *Brucella *strains developed bacteraemia, and the bacteria isolated from the blood matched one of the isolates she was working with, indicating a laboratory infection [56]. Two patients from Peru were presented with intracerebral granulomas and marine mammal brucellae were isolated from the lesions. Both had been at the coast and had eaten raw shellfish [57]. Marine mammal brucellae have also been isolated from a human case of spinal osteomyelitis in New Zealand. The patient had eaten fish and been in contact with raw fish bait [54]. There was no known contact with marine mammals for any of these three naturally acquired human cases. Characterisation [58] of the four marine mammal *Brucella *spp. isolated from humans [54,56,57] showed that the three strains from the naturally acquired infections differed from the strains isolated from marine mammals, and also from the strain isolated in the case of the laboratory acquired zoonotic marine mammal *Brucella *infection [58].

Experimental infection of human macrophage-like cells in culture has shown that *B. pinnipedialis *from harbour seal and *B. ceti *from striped dolphin showed the classical pattern of infection. However, another strain of *B. pinnipedialis *from harbour seal, and *B. ceti *from porpoise and common dolphin were eliminated from the infected cells after 48 h. Further, six strains of *B. pinnipedialis *from hooded seal were unable to enter the human macrophage-like cells [59].

The *Brucella *status of a country is based on the epidemiological situation in domestic animals and *B. ceti *and *B. pinnipedialis *are not considered. Thus, countries considered free of the disease [60] may have *Brucella *spp. present in its marine mammal populations [30,34]. Because of the varied symptoms of human brucellosis [14], and the very recent awareness of the existence of *Brucella *spp. in marine mammals [28,29], transmission from marine mammals to humans could historically have been reported as terrestrial brucellosis or, even more likely, have gone undetected. People at risk of acquiring marine mammal brucellosis include individuals in traditional communities where products from whales and seals are still an important part of the diet. Also people with limited consumption of marine mammal meat, people handling stranded marine mammals, whale and seal hunters, researchers and other people handling raw products from the ocean could be exposed.

## 3. Characterisation of *B. ceti *and *B. pinnipedialis*

Minimal standards for descriptions of new species and biotypes of genus *Brucella *were proposed in 1975 [61]. In 2003, the Subcommittee on the Taxonomy of *Brucella *reevaluated criteria for *Brucella *species definition to respond to developments within molecular techniques, especially focusing on the marine mammal strains with the suggested names *Brucella pinnipediae *and *Brucella cetaceae *[6,62]. Based on previous studies and recommendations [6,61-73], two novel species were validly published in 2007, labelled with corrected etymology, for inclusion in the genus *Brucella*; *Brucella ceti *sp. nov. and *Brucella pinnipedialis *sp. nov., with cetaceans and seals as their preferred hosts, respectively [7].

### 3.1. Classical typing approaches

A large study including 102 isolates of *B. ceti *and *B. pinnipedialis *suggested that there is substantial variation in biotyping characteristics for these strains, with the requirement of CO_2 _being especially useful since the seal isolates need CO_2 _to grow, and the cetacean isolates do not [74]. These characteristics were in line with previous findings of one marine mammal *Brucella *group consisting of isolates from pinnipeds (including an otter), and one cetacean group, based on their CO_2_-dependency [41,64] and ability to grow on Farrell medium in primary culture [41]. The oxidative metabolic pattern on different substrates supported this subdivision, with group one consisting of isolates from seals and otters, and group two and three consisting of cetacean isolates [71]. A commercial biotyping system (Taxa Profile™, Merlin Diagnostika, Bornheim-Hersel, Germany) testing the metabolization of various substrates also readily differentiated between the strains from common seal and porpoise with a specificity of 100% [75].

### 3.2. Identification of *B. ceti *and *B. pinnipedialis *as part of genus *Brucella*

Sequencing of the 16S rRNA from a minke whale *Brucella *isolate showed a 99.5% homology with the 16S rRNA of the six classical *Brucella *species, above the 97% homology that often is quoted as the cut-off between species [65]. Multiple sequence alignments of partial *recA *and 16S rRNA gene sequences of the six classical *Brucella *species and isolates from a whale and a common seal (erroneously reported as a sea-lion isolate in the article, changed after personal communication with the author) also showed that all strains were identical [68]. DNA-DNA hybridization studies showed that the marine mammal brucellae belong to the monospecific genus *Brucella *(more than 77% DNA relatedness) [63,76].

The 16S-23S ribosomal spacer region, the most variable region of the ribosomal genes for bacteria of the α-proteobacteria, has been used to identify a number of isolates [77,78]. However, some taxa having a high 16S rRNA similarity still show low DNA-DNA binding values [79], indicating that these methods might not accurately reflect the strains' phylogenetic status. There is also concern that single-gene trees, including those based on the 16S-23S ribosomal spacer region and housekeeping gene *recA*, do not adequately portray the phylogenetic relationships [80], and it has been suggested that at least five housekeeping genes should be sequenced to achieve adequate phylogenetic information [73].

### 3.3. Molecular characterisation: band-based approaches

Common for the band-based techniques is that they rely on cutting of the genome, or PCR amplicons, by restriction enzymes at specific sites, and the production of specific band patterns which can be compared to determine relatedness between isolates.

#### 3.3.1. Pulsed-Field Gel Electrophoresis (PFGE) and early Polymerase Chain Reaction (PCR) based approaches

PFGE was used in early studies [81,82], but the method was never widely used as a routine typing tool of *Brucella *spp., probably because of the limited diversity identified at a sub-species level [83]. In a study of brucellae from dolphins, porpoises and seals, PFGE indicated that the dolphin isolates clustered together, while the isolates from seals and porpoises clustered together in another main branch, separating at 57% relatedness [84]. A later PFGE study gave three similar groups; group one with isolates from seals and an otter, group two with strains from dolphins and group three isolates from porpoises, a white-sided dolphin and a minke whale [85]. A dendrogram based on IRS-PCR (Infrequent Restriction Site PCR) showed that the marine mammal isolates formed two groups distinct from the terrestrial strains; group one containing the pinniped isolates, and group two the cetacean isolates. The occurrence of a genomic island in the pinniped isolates was also documented [69]. The same IRS-PCR method later revealed five DNA fragments specific to the marine mammal strains. Two of the new DNA fragments were present in all seal isolates, except for the hooded seal isolates. The presence of genomic islands was indicated in these fragments [86]. Although AFLP (Amplified Fragment Length Polymorphism PCR) profiles were highly conserved through the genus, indicating limited intraspecies diversity, *B. ovis, B. melitensis, B. abortus, B. neotomae *and the marine mammal strains still fell into five separate clusters with linkage levels above 93%. The linkage level within the marine mammal group was 96%. This study confirmed the homogeneity of the genus and that the marine mammal strains belong to a separate branch of the genus [87]. RAPD-PCR (Random Amplification Polymorphic DNA PCR) was applied to a large number of terrestrial and marine mammal *Brucella *spp. and successfully separated the seal and cetacean strains. A 569-bp band was amplified from all seal strains and one porpoise strain. The amplification of a 217-bp band from the cetacean isolates was not consistent within the group, indicating two possible subdivisions [88]. The *Hind*III ribotyping restriction pattern from the six *Brucella *spp. reference strains and *Brucella *spp. from a harbour seal, a porpoise and a common dolphin showed that the marine mammal strains had the same restriction pattern, different from the pattern of the terrestrial strains. It was concluded that the marine mammal strains may represent a separate subgroup of the *Brucella *genus and that further studies were needed to define biovars within the group [76].

#### 3.3.2. Studies of the outer membrane protein genes (*omp*-genes)

PCR-RFLP (PCR Restriction Fragment Length Polymorphism) is a common approach for typing of *Brucella *spp., providing a good tool for taxonomic, epidemiological, evolutionary and diagnostic studies. The method has especially been utilized in studies of various outer membrane protein (*omp*) genes [89]. The *omp2a *and *omp2b *genes encode the 36-kDa porin OMPs and exhibit the highest degree of polymorphism among the *Brucella *species and strains [90]. PCR-RFLP of the *omp2 *locus has good reproducibility and has been useful for differentiation of *Brucella *species, even though it is somewhat limited by the lack of natural sequence differences at the biovar level. The *omp25 *and *omp31 *genes have been found useful for differentiation of species [62,91-93].

A *Brucella *isolate from a minke whale (B202R) was classified as belonging to the *Brucella *genus by conventional bacteriological typing methods, but it did not match any of the previously known profiles. The *omp25 *and *omp31 *genes were amplified from the isolate, indicating that it belonged to the genus [65], but none of the species-specific markers for the classical *Brucella *species [92-95] were found. PCR-RFLP and sequencing of the *omp2a *and *omp2b *genes showed that the isolate carried two copies of *omp2b*, but no copies of *omp2a*, a characteristic not previously seen in any other brucellae [65]. A later study confirmed that the marine mammal isolates were divided into two main groups; one group with isolates from cetaceans possessing two *omp2b *copies and one group with isolates from seals and an otter possessing one *omp2a *and one *omp2b *gene copy [62]. A DNA inversion of 1747 basepairs including the *omp25b *gene was detected in 16 of 20 cetacean isolates, but in none of the pinniped isolates. It was speculated that this suggests the existence of biovars within the marine mammal brucellae, but the findings nevertheless supported two separate marine mammal species [70].

#### 3.3.3. Studies of insertion sequence 711 (*IS*711)

The identification of the number and distribution of the mobile genetic element *IS*711 is an important tool for molecular characterisation of *Brucella *spp. even though some of the reference strains have identical profiles and no thorough studies evaluating this method have been published. The number of *IS*711 varies from 6-12 copies in most *Brucella *spp. to about 35 copies in *B. ovis *[83,96-98].

RFLP and southern blotting conducted with an *IS*711 specific probe on the previously mentioned B202R *Brucella *isolate from a minke whale, showed more than 25 copies of this sequence [65]. A later study confirmed that in the marine mammal isolates, *IS*711 occurred invariably with at least 25 copies. The ten different fingerprints found within the marine mammal group tended to cluster by host species. Using a specific marker, an *IS*711 locus not present in the terrestrial strains was identified in the marine mammal strains. It produced small amounts of PCR amplification products from *B. ovis*, suggesting a possible closer relationship between the marine mammal *Brucella *spp. and *B. ovis *[66]. An extra *IS*711 element downstream of the *bp26/omp28 *gene has been identified in marine mammal brucellae as a specific marker for the marine mammal strains [67], and later included in a multiplex PCR assay ("Bruceladder") [99,100].

### 3.4. Molecular characterisation: sequence-based approaches

Since the first whole genome sequence publications for *Brucella *spp. [101-103], a wide range of species and strains have been sequenced and are available online [104]. This information has enabled the progressive shift from band-based to sequence-based approaches. Previous studies have indicated that inactivation of genes may result in the lack of pathogenicity of *B. ovis *[105], and that several genes important for pathogenicity may have entered the *Brucella *genome by lateral gene transfer, despite their intracellular niche [106]. This highlights the need for full genome analysis methods.

#### 3.4.1. Analysis based on tandem repeats

The identification of the number of tandemly repeated sequences is useful for discrimination between bacterial species that show very little genomic variability like *Brucella *spp. Microsatellite fingerprinting exploits the occurrence of Variable Number of Tandem Repeats (VNTR) in the genome. When considering multiple loci simultaneously, the method achieves high discriminatory power. With the appropriate choice of markers the method yields information about both the faster (epidemiological level) and the slower (phylogenetic level) evolving genes [83,107-109], though some caution should be made regarding the rapidly evolving VNTR markers which may suffer from homoplasy (the same alterations happening in several branches of a phylogenetic tree), thus preventing accurate speciation of some isolates [110]. The results are digital, making inter-laboratory cooperation easier, and an online database with *Brucella *spp. fingerprints is available [111].

Maximum parsimony analysis after Multiple Loci VNTR Analysis (MLVA) using 15 discriminatory markers showed that the marine mammal isolates of *Brucella *were distinct from terrestrial isolates. The three marine mammal isolates were different from each other with the isolates from dolphin and porpoise more closely related, and the harbour seal isolate clustering closer to *B. suis *[109]. Another dendrogram produced from MLVA indexing 21 loci showed three major groups of marine mammal strains, distinct from the terrestrial strains. Cluster A consisted only of dolphin isolates and cluster B of mainly isolates from porpoises. Cluster C consisted mostly of seal isolates which were further divided into three minor groups. The authors concluded that cluster C corresponded to *B. pinnipedialis*, while isolates previously categorized within *B. ceti *were divided into two groups with isolates from dolphins in one group and porpoises in the other. These findings were confirmed by sequencing of eight additional housekeeping genes from all isolates [112]. An MLVA-16 assay [8-10,108,109,113-115] also revealed a very similar grouping with three main marine mammal *Brucella *groups; the seal group (1) including an isolate from a sea otter, the cetacean group (2) and a human isolate (3). The seal isolates (1) were divided into three distinct subclusters, with the hooded seal isolates grouped together in one subcluster. The cetacean group (2) had three major subclusters, two with mostly dolphin isolates and one with mostly porpoises [116]. The highly discriminatory nature of the microsatellite fingerprinting suggests a subdivision between the strains from seals, porpoises and dolphins, and indicates that the hooded seal strains cluster separately.

#### 3.4.2. Multi locus sequence analysis (MLSA)

In recent years, sequencing of multiple genetic loci, often housekeeping genes with few polymorphic sites, has gained acceptance. Combined sequencing of multiple housekeeping genes allows conclusions to be based on multiple loci, making them more representative for the evolutionary development of the strain. The evolutionary signatures of the genes are preserved due to the markers used which in general have slow molecular clocks yielding information suited to monitor the long-term evolutionary development of the strains [83,117]. Another advantage of nucleotide sequencing is that both protocols and primers are easily acquired and the results are easily validated, stored and shared electronically, facilitating inter-laboratory comparison of results.

MLSA was conducted using nine discrete genomic loci on several terrestrial and marine mammal *Brucella *isolates, and one marine mammal strain [117] isolated from a human [54]. In clustering analysis, marine mammal isolates represented one cluster with five sequence types (STs): ST24 and ST25 with mostly seal isolates (80%), ST23 with mostly porpoises (75%), ST26 with only dolphins and ST27 with one bottlenose dolphin isolate and a human isolate. The authors concluded that the marine mammal isolates were so similar that they could be classified as a single species, but based on the genetic separation and the apparent host specificity they could also be three distinct species (*Brucella *spp. from seals, porpoises and dolphins) [117]. A larger MLSA study [112], utilizing the same genomic loci [117], showed the same pattern [112]. Similar to terrestrial strains [117], the MLSA method is useful for differentiation between and characterisation of marine mammal brucellae. A high degree of similarity between the strains was confirmed, a possible subdivision of the marine mammal brucellae was indicated (strains from seals, porpoises and dolphins) [112,117] and the hooded seal isolates clustered together in a separate subgroup [112].

#### 3.4.3. Single-Nucleotide Polymorphism (SNP) typing

The SNPs are discovered utilising MLSA of housekeeping genes or whole genome comparisons. The slowly evolving SNPs are used to define the major groups, and the more rapidly evolving VNTR markers are thereafter used to give higher resolution, yielding genotyping that can be used for epidemiological investigations. The increased level of genetic diversity in VNTRs compared to SNPs is based on differences in mutation rates but also because of the maximum number of possible allelic states for each type of marker [107,118]. The method is quick, technically straightforward and applicable to crude DNA-material. The method has shown to be able to identify any *Brucella *isolate as a member of the six classical species or the marine mammal group. However, it has not been possible to include SNP-markers to identify the new species *B. pinnipedialis *and *B. ceti *[119-121].

#### 3.4.4. Whole genome comparison

Taxonomic classification of *Brucella *spp. is difficult due to the lack of, or high degree of, similarity between traditional marker genes [122,123]. The marker genes may not directly reflect the change in gene content between strains and biovars [123]. Base compositional (Markov chain based genomic signatures, genomic codon and amino acid frequencies based comparison) and proteome based (BLAST comparison and pan-genomic analysis) methods are used for phylogenetic classification, based on whole genome comparison. By utilising whole genome based methods, one avoids drawing conclusions on only limited parts of the genome [124]. Base compositional and proteome based methods were used to compare 32 sequenced genomes from the *Brucella *genus, representing the six classical species, *B. ceti *and *B. pinnipedialis*. The codon and amino acid frequencies based comparison made no distinction between the terrestrial and marine mammal strains, confirming the homogeneity of the genus. The Markov chain based models grouped the species according to host, while signatures from the marine mammal isolates clustered together and could be considered as one phylogenetically coherent group. Whole genome BLAST (Basic Local Alignment Search Tool; finds regions of local similarity between sequences) comparisons were performed pairwise all-against-all and a distance matrix was calculated. The marine mammal strains clustered together due to similarity [124]. The findings were consistent with current taxonomy, indicating that the phylogenetic classification of *Brucella *spp. based on MLSA and marker genes [117] had a high similarity to the results achieved with the methods utilizing the whole gene content of the *Brucella *species. The pan-genomic shell trees, weighting shell (conserved) and cloud (variable) genes, both clustered the marine mammal strains together with a branching between the pinniped and the cetacean strains, but with low bootstrap support, indicating that the differences were negligible. The study confirmed the high level of homogeneity of the genus *Brucella *with a possible subdivision between seals and cetaceans. The hooded seal strain differed from the other strains when utilizing both base compositional and proteome based methods [124].

## 4. *B. pinnipedialis *in hooded seals

### 4.1. The biology of the hooded seal

Hooded seals (family *Phocidae*, subfamily *Phocinae*, the only species in genus *Cystophora*) are 2.2-2.5 meters long and weigh 200-300 kg [125,126]. Hooded seals are specialised divers, reaching depths of about 1000 meters and staying under water for up to one hour [127]. The hooded seal gives birth to a single pup and the lactation period (and the mother-pup relationship) is extremely short, lasting for about 3-5 days [128]. Mating takes place in the water immediately after weaning and implantation is delayed by 3-4 months. During birth (March) and moulting (June/July), hooded seals are gathered on the pack ice and during the rest of the year they are pelagic, distributed throughout the North Atlantic Ocean [126,129]. They feed primarily on squid (*Gonatus fabricii*), polar cod (*Boreogadus saida*), capelin (*Mallotus villosus*) and sand eel (*Ammodytes tobianus*) [130]. The hooded seal form two populations, the Northeast and the Northwest Atlantic population [126]. The Northeast Atlantic population has declined but stabilised at a level only 10-15% of what it was in 1946 [131]. Estimates of pup production suggested a production of 15-16 000 pups in 2005 and 2007, giving a stipulated population of 82 400 animals [132,133]. The Norwegian commercial hooded seal hunt on this population has been regulated by quotas during the last 25 years, and the hunting was stopped in 2007. The number of hooded seals hunted annually has declined and has not exceeded 10 000 since 1987 [131]. The situation for the Northwest Atlantic hooded seal population is very different where both the population size and the pup production have increased since the mid-1980s. Estimated pup production in 2005 was 120 100 and the population was stipulated to consist of 593 500 individuals [134,135]. The commercial hooded seal hunt on the Northwest Atlantic hooded seal population (Canada) has also been regulated for the last 25 years with approximately 6 000-7 000 animals hunted annually since 1972 [131]. The reason for the difference in population development is unknown, but the decline in the eastern stock has been so dramatic that the hooded seal species is classified as vulnerable in the International Union for Conservation of Nature (IUCN) Red List of Threatened Species [136].

### 4.2. Infection in hooded seals with *B. pinnipedialis*

Isolations of *B. pinnipedialis *from hooded seals have been conducted from either stranded animals [35,41] or from apparently healthy individuals hunted in their natural environment [30]. In the various studies on the characterisation and species and biovar determination referred to in this article, the hooded seal isolates are repeatedly classified as one group, distinct from other *B. pinnipedialis *isolates.

Despite the high level of seropositive (31-35%) [30,34] and bacteriological positive (38%) [30] hooded seals in the Northeast Atlantic population, no associated gross pathological changes have been reported [30,34]. A large screening for anti-*Brucella *antibodies in the Northwest Atlantic hooded seal population gave a seroprevalence of 5% (*n *= 10/204). These animals were not investigated for pathological changes [137]. Based on the restrictive quotas and the low number of hunted seals it seems unlikely that the large difference in population development can be due to seal hunting alone. Climatic changes and poor ice quality probably have an impact on the quality of the breeding habitat, but it is unlikely that only the Northeast Atlantic population is affected. Other possible factors include persistent organic pollutants (POP) or infectious diseases affecting reproduction, survival and general health and fitness of the seals. Based on available information, it is not possible to determine if the high prevalence of *B. pinnipedialis *in the Northeast Atlantic population of hooded seals, associated with POP exposure, may explain the decline in pup production, an issue which warrants further investigation.

### 4.3. Characterisation of the hooded seal strains of *B. pinnipedialis*

In a comparative study, a hooded seal isolate was the only *Brucella *isolate to have metabolic activity with urocanic acid [64]. PFGE showed that the *Brucella *isolate from hooded seal lacked a 182 kB fragment and had some minor differences in a 62 kB fragment, thereby diverging from the other seal isolates [85]. IRS-PCR showed two specific DNA fragments present in all isolates from seals, except the hooded seal isolates. It was suggested that these fragments could be part of metabolic genomic islands. Their absence could be a result of hooded seal isolates being an ancestor inside the *B. pinnipedialis *species [86]. Studies of DNA polymorphism at the *omp2 *locus showed that the hooded seal isolate was classified in a separate group. Of special interest was the *Alu*I restriction pattern of *omp2b*, which was identical for all marine mammal isolates except the hooded seal isolate [62]. The results from VNTR and MLSA on the same isolates were coherent with each other and showed that the hooded seal isolates clustered in a separate subgroup within the pinniped group [112]. MLVA on 15 isolates from hooded seals gave a clustering in 9 closely related genotypes within the pinniped cluster [116]. Whole genome comparison by Markov chain based methods placed the hooded seal isolate in a separate subgroup and indicated large base compositional differences between this isolate and the other *Brucella *spp., including the marine mammal isolates. The pan-genomic analysis showed that the hooded seal isolate differed from all other brucellae. The hooded seal genome was also the most GC rich of all of the analysed genomes suggesting that the hooded seal isolate might be closely related to an unknown ancestor of *Brucella *spp. [124]. The differences between isolates of *Brucella *spp. from hooded seals and other marine mammals are summarised in Table 1.

**Table 1:** Differences between isolates of *Brucella *spp. from hooded seals and other marine mammals.

Methods	Results	Reference
Biotyping and metabolic activity	The only *Brucella *isolate to metabolize urocanic acid.	[64]

PFGE	The hooded seal isolate lacked a 182 kB fragment and had some minor differences in a 62 kB fragment which was specific for the pinniped strains.	[85]

IRS-PCR	There were two specific DNA fragments (fragments 2 and 3) present in all isolates from seals, except the hooded seal isolates. These fragments might be part of metabolic genomic islands. Their absence suggests that the hooded seal isolates may be closely related to an unknown ancestor of *Brucella *spp.	[86]

PCR-RFLP studies of DNA polymorphism at the *omp2 *locus	The hooded seal isolate was classified in a separate group. The *Alu*I restriction pattern of *omp2b *was identical for all marine mammal isolates except the hooded seal isolate.	[62]

VNTR/MLVA	Both VNTR and MLVA subclustered the hooded seal isolates in a separate subcluster (C3), within the *B. pinnipedialis *cluster.	[112,116]

MLSA	The hooded seal isolates belonged to ST25, corresponding to the C3 subcluster mentioned above.	[112]

Whole genome comparison by Markov chain based methods	The hooded seal isolate grouped separately indicating relatively large genomic compositional differences between this isolate and other brucellae.	[124]

Pan-genomic analysis	The hooded seal isolate differed from all other *Brucella *spp. in gene content. The hooded seal genome was also the most GC rich of all the analysed genomes, suggesting that the hooded seal isolate might be closely related to an unknown ancestor of *Brucella *spp.	[124]

Experimental infection of human macrophage-like cells in culture	The six hooded seal isolates were unable to enter the human macrophage-like cells.	[59]

## 5. Conclusions and future perspectives

Biotyping is time consuming and requires handling of live brucellae. The number of characteristics defining a biovar varies, the interpretation of results can be rather subjective, requiring expertise and experience and some strains can yield unexpected results. Although bacterial isolation and biotyping are the gold standards [138], these methods have to be complemented with molecular studies to differentiate between the different *Brucella *spp. [41,64,71,74].

Chromosomal rearrangements and gain/loss of restriction sites and/or primer binding sites have a great impact on the banding patterns produced by PGFE [80,139] and PCR-based methods [140]. The band-based approaches in general lack the ability to discriminate at a sub-species level and they pose a challenge when it comes to inter-laboratory reproducibility [83,141-144]. Although several useful discriminatory markers for *Brucella *spp. have been identified by these methods, they have limited applications for molecular characterisation of the marine mammal brucellae.

Due to the limitations of the band-based methods and the increasing availability of genome sequences, there has been a gradual shift from band-based to sequence-based approaches; namely MLVA, MLSA, SNP-typing and whole genome analysis techniques. The sequence-based approaches generate data that are easily stored and shared electronically, making the development of international generic databases possible, and facilitating international cooperation. By choosing genetic markers with slow or fast molecular clocks one can decide at what level one gains information, ranging from local epidemiological investigations to phylogeny analysis [83].

Several studies have indicated that the hooded seal isolates form one group which differs from other isolates obtained from seals (Summarised in Table 1) [62,64,85,86,112,116,124]. Differentiation between *Brucella *spp. isolated from porpoises and dolphins have also been indicated in several studies [84,85,112,116,117]. This division is also in accordance with the classical taxonomy based on host specificity [83].

Marine mammal *Brucella *spp. are not a uniform group when it comes to zoonotic potential. Only some marine mammal *Brucella *isolates seem to be able to infect humans, and further work on these isolates is needed in order to characterise their zoonotic potential.

The pathogenicity of *B. pinnipedialis *in seals, and hooded seals in particular, is unclear, and the impact this infection has on the Northeast Atlantic hooded seal population, having a decline in pup production, warrants further investigation. Gross pathology induced by *Brucella *infection in marine mammals is only seen in cetaceans, supporting a difference either in the pathogenicity of the marine mammal *Brucella *spp. or in the susceptibility of cetaceans as opposed to pinnipeds. Histopathology studies are needed in order to gain insight into the pathogenicity of strains in their preferential hosts.

It has been suggested that lungworms carrying *Brucella *spp. are the means by which marine mammals become infected [36,45,46,48]. Pinnipeds are infected with lungworms by consumption of intermediate host fish species. *Parafilaroides decorus *in the California sea lion uses a tide pool fish (*Girella nigricans*) as an intermediate vertebrate host [145,146], whereas *P. gymnurus *larvae and *Pseudalius inflexus *were isolated from plaice *(Platessa pleuronectes*) and dab (*Limanda limanda*) [147]. Whether the *Brucella *infection originates from fish or from fish parasites is unknown and warrants further investigation.

Since the *Brucella *isolates from hooded seals are clearly diverging from other seal isolates [62,64,85,86,112,116,124] and because the hooded seal biology (pelagic life, deep divers, short mother-pup relationship) is different from that of many other seal species [126-129], further work is needed to characterise these isolates and to address the impact that the infection may have on hooded seals, both on individuals and at the population level. This is especially important, since the Northwest Atlantic population, which has a high prevalence of *B. pinnipedialis *infection [30,34], has declined [131-133], and the whole hooded seal species is classified as vulnerable in the IUCN Red List of Threatened Species [136].

Contaminants accumulate in the Arctic regions, and very high levels, especially of polychlorinated biphenyls (PCB), have been reported in top predators and in the environment [148-150]. Climate change may affect the contamination pathways so that we may see an increased level of POPs in the Arctic regions in the future [149,151]. Several studies have indicated that exposure to POPs leads to inadequate immune function and higher risk of disease development [149,152-154]. Whether high levels of POPs affect the hooded seals to such a degree that an infection with *B. pinnipedialis *can develop into clinical disease, impacting hooded seal population dynamics, warrants further investigation.

## Competing interests

The authors declare that they have no competing interests.

## Authors' contributions

IHN did the main work with the manuscript. JG and MT gave advice in drafting the manuscript and revised it critically. All authors read and approved the final manuscript.

## Supplementary Material

Additional file 1 **Isolation of *Brucella *spp. from marine mammals**. Overview of literature describing the isolation of *Brucella *spp. from marine mammals, the prevalence of bacteriological positive animals, and the organ of origin for isolation and pathology. Click here for file

Additional file 2 **Serological evidence of *Brucella *spp. in marine mammals**. Overview of literature describing the presence and seroprevalence of anti-*Brucella *antibodies in marine mammal species. Click here for file
