# Supplementary material for: A review of Brucella infection in marine mammals, with special emphasis on Brucella pinnipedialis in the hooded seal (Cystophora cristata)
Source: Vet Res. 2011 Aug 5;42(1):93. doi: 10.1186/1297-9716-42-93 (PMC3161862; doi:10.1186/1297-9716-42-93)
Supplement: Additional file 1 — Isolation of Brucella spp. from marine mammals. Overview of literature describing the isolation of Brucella spp. from marine mammals, the prevalence of bacteriological positive animals, and the organ of origin for isolation and pathology. [file 1297-9716-42-93-S1.DOCX]

**Additional file 1. Isolation of *Brucella* spp. from marine mammals.**

| **Species** | | **Organ of origin and pathology associated** | **Prevalence** | **Reference** |
| --- | --- | --- | --- | --- |
| Hooded seal (*Cystophora cristata*) | | Highest tissue prevalence in spleen and lung lymph nodes. No pathology associated. | 11/28 (38 %) | [1] |
|  |  | From several different organs. One pup with sheets of macrophages in the spleen, otherwise no evidence of *Brucella*-associated pathology. | 3/3 (100 %) | [2,3] |
| Ringed seal (*Pusa hispida*) | | From pooled lymph nodes of seropositive animals. No pathology associated. | 4/6 (67 %) | [4] |
| Harp seal (*Pagophilus groenlandicus*) | | From lungs and lymph nodes. No pathology associated. | 3/9 (33 %) | [5] |
|  |  | From pooled lymph nodes from a seropositive animal. No pathology associated. | 1/1 (100 %) | [4] |
| Grey seal (*Halichoerus grypus*) | | From lung tissue in one animal. No pathology associated. | 1/34 (3 %) | [6] |
|  |  | From the testes of one animal. No pathology associated. | 1/1 (100 %) | [2] |
| Pacific harbour seal (*Phoca vitulina richardii*) | | From multiple lymph nodes and lungs of one stranded, dead animal, no pathology associated. Evidence of *Brucella* spp. in Parafilaroides lungworms, pathology in lungs typical of this. | 1/1 (100 %) | [7] |
| Harbour seal (common seal) (*Phoca vitulina*) | | From several internal organs of animals found dead. No pathology associated. | 4/18 (22 %) | [8] |
|  |  | From lungs and lymph nodes. No pathology associated. | 2/4 (50 %) | [5] |
|  |  | Mostly from lungs and lung lymph nodes. Also from digestive tract, spleen, kidneys and liver. No pathology associated. | 47/426 (11 %) | [6] |
|  |  | From spleen and several lymph nodes. No pathology associated. | 7/7 (100 %) | [2,9,10] |
| California sea lion (*Zalophus* *californianus*) | From two placentas. No *Brucella*-pathology associated. | 2/67 (3 %) | [11] |  |
| Harbour porpoise (*Phocoena phocoena*) | From several tissues and the lungworms of a stranded, dead animal. No pathology associated besides hyperaemic lungs. | 1/1 (100 %) | [12] |  |
|  | From subcutaneous lesions in all four animals, also from spleen, mandibular lymph nodes, blood and uterus in some of the animals. No other pathology associated than the subcutaneous lesions. | 4/4 (100 %) | [2,9,10] |  |
|  | From brain and lung tissue of a stranded animal. Emaciation, skin ulcers, pneumonia, necrotic foci in liver, meningitis, endometritis. | 1/1 (100 %) | [13] |  |
|  | From multiple organs and lungworms of an animal who had died from entanglement in a fishing net. Hyperaemic lungs. | 1/1 (100 %) | [14] |  |
|  | From lung, kidney and mesenteric lymph nodes. No pathology associated. | 2/298 (1 %) | [6] |  |
| Common dolphin *(Delphinus delphis*) | From subcutaneous lesions in one stranded animal. | 1/1 (100 %) | [2,9,10] |  |
| Striped dolphin (*Stenella coeuleoalba*) | From the brain and blood of a live-stranded dolphin. Meningoencephalitis present. | 1/1 (100 %) | [15] |  |
|  | From the brain and several other organs in stranded animals. Meningoencephalomyelitis present in all nine animals. Placentitis and a dead infected foetus in the pregnant animal. | 8/9 (89 %) | [16] |  |
|  | From the central nervous system and all organs tested in a live-stranded animal. Multiple pathological changes in the central nervous system. | 1/1 (100 %) | [17] |  |
|  | From the brain of three young, stranded, animals found dead, all with meningoencephalitis. | 3/3 (100 %) | [18] |  |
|  | From the cerebrospinal fluid of six stranded animals and in the reproductive organ and foetus of a pregnant animal. Meningoencephalitis was present in all ten animals. | 6/10 (60 %) | [19] |  |
|  | The highest number of colonies came from subcutaneous lesions. Several other positive organs. | 2/2 (100 %) | [2] |  |
| Atlantic white-sided dolphin (*Lagenorhynchus acutus*) | From a diseased atlanto-occipital joint, the brain and a kidney from one dead-stranded animal. Meningoencephalitis present. | 1/1 (100 %) | [20] |  |
|  | From the gastric lymph node of one animal. No pathology associated. | 1/1 (100 %) | [2] |  |
| Bottlenose dolphin (*Tursiops truncatus*) | From two aborted foetuses and from vaginal fluids from one mother animal. Placentitis present in both cases. Isolation also from lung necropsy of another animal at the same location. | 4/4 (100 %) | [21] |  |
|  | From a purulent abscess within the blubber of a wild bottlenose dolphin. | 1/1 (100 %) | [22] |  |
|  | From an aborted foetus of a bottlenose dolphin in captivity. | 1/1 (100 %) | [23] |  |
| Killer whale (*Orcinus orca*) | From the reproductive tissues of a stranded killer whale. | 1/5 (20 %) | [24] |  |
| Minke whale (*Balaenoptera acutorostrata*) | From the liver and spleen of a seropositive animal. No pathology associated. | 1/7 (14 %) | [25] |  |
| European otter (*Lutra lutra*) | From the internal iliac lymph node of an otter killed in a road traffic accident. No evidence of *Brucella*-associated pathology. | 1/1 (100 %) | [2] |  |

Reference List

1. Tryland M, Sorensen KK, Godfroid J: **Prevalence of *Brucella* *pinnipediae* in healthy hooded seals (*Cystophora cristata*) from the North Atlantic Ocean and ringed seals (*Phoca hispida*) from Svalbard.** *Vet Microbiol* 2005, **105:**103-111.

2. Foster G, Jahans KL, Reid RJ, Ross HM: **Isolation of *Brucella* species from cetaceans, seals and an otter.** *Vet Rec* 1996, **138:**583-586.

3. Foster G, MacMillan AP, Godfroid J, Howie F, Ross HM, Cloeckaert A, Reid RJ, Brew S, Patterson IAP: **A review of *Brucella* sp infection of sea mammals with particular emphasis on isolates from Scotland.** *Vet Microbiol* 2002, **90:**563-580.

4. Forbes LB, Nielsen O, Measures L, Ewalt DR: **Brucellosis in ringed seals and harp seals from Canada.** *J Wildl Dis* 2000, **36:**595-598.

5. Maratea J, Ewalt DR, Frasca S, Dunn JL, De Guise S, Szkudlarek L, St Aubin DJ, French RA: **Evidence of *Brucella* sp. infection in marine mammals stranded along the coast of southern New England.** *J Zoo Wildl Med* 2003, **34:**256-261.

6. Prenger-Berninghoff E, Siebert U, Stede M, Koenig A, Weiss R, Baljer G: **Incidence of *Brucella* species in marine mammals of the German north sea.** *Dis Aquat Organ* 2008, **81:**65-71.

7. Garner MM, Lambourn DM, Jeffries SJ, Hall PB, Rhyan JC, Ewalt DR, Polzin LM, Cheville NF: **Evidence of *Brucella* infection in Parafilaroides lungworms in a Pacific harbor seal (*Phoca vitulina richardsi*).** *J Vet Diagn Invest* 1997, **9:**298-303.

8. Watson CR, Hanna R, Porter R, McConnell W, Graham DA, Kennedy S, McDowell SWJ: **Isolation of *Brucella* species from common seals in Northern Ireland.** *Vet Rec* 2003, **153:**155-156.

9. Ross HM, Jahans KL, MacMillan AP, Reid RJ, Thompson PM, Foster G: ***Brucella* species infection in North Sea Seal and cetacean populations.** *Vet Rec* 1996, **138:**647-648.

10. Ross HM, Foster G, Reid RJ, Jahans KL, MacMillan AP: ***Brucella* Species Infection in Sea-Mammals.** *Vet Rec* 1994, **134:**359.

11. Goldstein T, Zabka TS, Delong RL, Wheeler EA, Ylitalo G, Bargu S, Silver M, Leighfield T, Van Dolah F, Langlois G et al.: **The Role of Domoic Acid in Abortion and Premature Parturition of California Sea Lions (*Zalophus Californianus*) on San Miguel Island, California.** *J Wildl Dis* 2009, **45:**91-108.

12. Perrett LL, Dawson CE, Davison N, Quinney S: ***Brucella* infection of lungworms from a harbour porpoise.** *Vet Rec* 2004, **154:**800.

13. Jauniaux TP, Brenez C, Fretin D, Godfroid J, Haelters J, Jacques T, Kerckhof F, Mast J, Sarlet M, Coignoul FL: ***Brucella ceti* Infection in Harbor Porpoise.** *Emerg Infect Dis* 2010, **16**.

14. Dawson CE, Perrett LL, Stubberfield EJ, Stack JA, Farrelly SSJ, Cooley WA, Davison NJ, Quinney S: **Isolation and characterization of *Brucella* from the lungworms of a harbor porpoise (*Phocoena phocoena*).** *J Wildl Dis* 2008, **44:**237-246.

15. Davison NJ, Cranwell MP, Perrett LL, Dawson CE, Deaville R, Stubberfield EJ, Jarvis DS, Jepson PD: **Meningoencephalitis associated with *Brucella* species in a live-stranded striped dolphin (*Stenella coeruleoalba*) in south-west England.** *Vet Rec* 2009, **165:**86-89.

16. Gonzalez-Barrientos R, Morales JA, Hernandez-Mora G, Barquero-Calvo E, Guzman-Verri C, Chaves-Olarte E, Moreno E: **Pathology of Striped Dolphins (*Stenella coeruleoalba*) Infected with *Brucella ceti*.** *J Comp Pathol* 2010, **142:**347-352.

17. Munoz PM, Garcia-Castrillo G, Lopez-Garcia P, Gonzalez-Cueli JC, De Miguel MJ, Marin CM, Barberan M, Blasco JM: **Isolation of *Brucella* species from a live-stranded striped dolphin (*Stenella coeruleoalba*) in Spain.** *Vet Rec* 2006, **158:**450-451.

18. Gonzalez L, Patterson IA, Reid RJ, Foster G, Barberan M, Blasco JM, Kennedy S, Howie FE, Godroid J, MacMillan AP et al.: **Chronic meningoencephalitis associated with *Brucella* sp. infection in live-stranded striped dolphins (*Stenella coeruleoalba*).** *J Comp Pathol* 2002, **126:**147-152.

19. Hernandez-Mora G, Gonzalez-Barrientos R, Morales JA, Chaves-Olarte E, Guzman-Verri C, Baquero-Calvo E, De-Miguel MJ, Marin CM, Blasco JM, Moreno E: **Neurobrucellosis in Stranded Dolphins, Costa Rica.** *Emerg Infect Dis* 2008, **14:**1825.

20. Dagleish MP, Barley J, Howie FE, Reid RJ, Herman J, Foster G: **Isolation of *Brucella* species from a diseased atlanto-occipital joint of an Atlantic white-sided dolphin (*Lagenorhynchus acutus*).** *Vet Rec* 2007, **160:**876-878.

21. Miller WG, Adams LG, Ficht TA, Cheville NF, Payeur JP, Harley DR, House C, Ridgway SH: ***Brucella*-induced abortions and infection in bottlenose dolphins (*Tursiops truncatus*).** *J Zoo Wildl Med* 1999, **30:**100-110.

22. Dawson CE, Perrett LL, Young EJ, Davison NJ, Monies RJ: **Isolation of *Brucella* species from a bottlenosed dolphin (*Tursiops truncatus*).** *Vet Rec 2006*, **158:**831-832.

23. Ewalt DR, Payeur JB, Martin BM, Cummins DR, Miller WG: **Characteristics of A *Brucella* Species from A Bottle-Nosed-Dolphin (*Tursiops-Truncatus*).** *J Vet Diagn Invest* 1994, **6:**448-452.

24. Raverty SA, Gaydos JK, Nielsen K, Nielsen O, Ross PS, Lambourn DM, Jeffries SJ: **An Overview of Marine Mammal Diagnoses in the Pacific Northwest from 1999 to 2004 .** In *World Small Animal Veterinary Association World Congress: 8-11 August 2002; Vancouver, Canada.*

25. Tryland M, Kleivane L, Alfredsson A, Kjeld M, Arnason A, Stuen S, Godfroid J: **Evidence of *Brucella* infection in marine mammals in the North Atlantic Ocean.** *Vet Rec* 1999, **144:**588-592.
