# Supplementary material for: A review of Brucella infection in marine mammals, with special emphasis on Brucella pinnipedialis in the hooded seal (Cystophora cristata)
Source: Vet Res. 2011 Aug 5;42(1):93. doi: 10.1186/1297-9716-42-93 (PMC3161862; doi:10.1186/1297-9716-42-93)
Supplement: Additional file 2 — Serological evidence of Brucella spp. in marine mammals. Overview of literature describing the presence and seroprevalence of anti-Brucella antibodies in marine mammal species. [file 1297-9716-42-93-S2.DOCX]

**Additional file 2. Serological evidence of *Brucella* spp. in marine mammals.**

| **Species** | **Prevalence** | **Reference** |
| --- | --- | --- |
| Hooded seal (*Cystophora cristata*) | 9/29 (31 %) | [1] |
|  | 48/137 (35 %) | [2] |
|  | 10/204 (5 %) | [3] |
| Ringed seal (*Pusa hispida*) | 5/49 (10 %) | [2] |
|  | 10/248 (4 %) | [4] |
|  | 7/628 (1 %) | [3] |
| Harp seal (*Pagophilus groenlandicus*) | 15/811 (2 %) | [2] |
|  | 8/453 (2 %) | [3] |
|  | 4/53 (8 %) | [5] |
| Grey seal (*Halichoerus grypus*) | 4-10/31 (13-32 %)* | [6,7] |
|  | 10/255 (4 %) | [3] |
|  | 6/62 (10 %) | [8] |
| Pacific harbour seal (*Phoca vitulina richardii*) | 1/1 (100 %) | [9] |
|  | 46/100 (46 %) | [10] |
| Harbour seal (common seal) (*Phoca vitulina*) | 25-69/140 (18-49 %)* | [6,7] |
|  | 21/163 (13 %) | [3] |
|  | 1/12 (8 %) | [8] |
|  | 3/21 (14 %) | [5] |
|  | (7-54 %)** | [11] |
|  | 175/1250 (14 %) | [12] |
| Atlantic walrus (*Odobenus rosmarus rosmarus)* | 7/59 (12 %) | [4] |
|  | 5/170 (3 %) | [3] |
| Steller sea lion (*Eumetopias jubatus*) | 1/197 (1 %) | [13] |
| Hawaiian monk seal (*Monachus schauinslandi*) | 19-28/144 (12-19 %)* | [14] |
| Wedell seal (*Leptonychotes weddellii*) | (5-33 %)* | [15] |
|  | 5/12 (42 %) | [16] |
|  | 0-1/1 (0-100 %)* | [17] |
| Antarctic fur seal (*Arctocephalus gazella*) | 1-5/16 (6-31 %)* | [17] |
|  | 4/65 (6 %) | [18] |
|  | 1-5/86 (1-6 %)* | [19] |
| Australian fur seal (*Arctocephalus pusillus doriferus*) | 88/304 (29 %) – 118/179 (66 %)* | [20] |
|  | 1/15 (7 %) | [21] |
| Australian sea lion (*Neophoca cinerea*) | 9/12 (75 %) | [21] |
| Leopard seal (*Hydrurga leptonyx*) | 1/3 (33 %) | [21] |
| Harbour porpoise (*Phocoena phocoena*) | 2-5/18 (11-28 %)* | [6,7] |
|  | 2/170 (1 %) | [22] |
|  | 11/35 (31 %) | [8] |
|  | 1/1 (100 %) | [23] |
|  | 9/23 (39 %) | [24] |
| Dall's porpoises (*Phocoenoides dalli*) | 2/6 (33 %) | [24] |
| Common dolphin *(Delphinus delphis*) | 1/1 (100 %) | [6,7] |
|  | 9/29 (31 %) | [8] |
|  | 1/2 (50 %) | [24] |
| Dusky dolphin (*Lagenorhynchus obscurus*) | 21/27 (78 %) | [25] |
| Long-beaked common dolphin (*Delphinus capensis*) | 3/6 (50 %) | [25] |
| Atlantic spotted dolphin (*Stenella frontalis*) | 0-1/1 (0-100 %)* | [26] |
| Striped dolphin (*Stenella coeuleoalba*) | 9-11/12 (75-92 %)* | [26] |
|  | 10/10 (100 %) | [27] |
|  | 2/2 (100 %) | [28] |
|  | 2/16 (13 %) | [25] |
|  | 1/1 (100 %) | [8] |
|  | 14/14 (100 %) | [29] |
|  | 1/1 (100 %) | [30] |
|  | 1/1 (100 %) | [31] |
| Clymene dolphin (*Stenella clymene*) | 0-1/6 (0-17 %)* | [26] |
| Bottlenose dolphin (*Tursiops truncatus*) | 7-31/55 (13-60 %)* | [26] |
|  | 4/7 (60 %) | [25] |
|  | 1/1 (100 %) | [8] |
|  | 1/1 (100 %) | [32] |
|  | 3/165 (2 %) | [33] |
| Black Sea Bottlenose dolphin (*Tursiops truncatus ponticus)* | 15-23/59 (25-39 %) | [34] |
|  | 17/74 (23 %) | [35] |
| Pacific bottlenose dolphin (*Tursiops aduncus*) | 31-40/58 (53-69 %)* | [36] |
| Burmeister's porpoise (*Phocoena spinipinnis*) | 5/25 (25 %) | [25] |
| Fraser's dolphin (*Lagenodelphis hosei*) | 2-13/24 (8-54 %)* | [26] |
| Northern right whale dolphin (*Lissodelphis borealis*) | 1/1 (100 %) | [24] |
| Rough-toothed dolphin (*Steno bredanensis*) | 5-14/23 (22-61 %)* | [26] |
| Risso's dolphin (*Grampus griseus*) | 6-8/11 (55-73 %)* | [26] |
| Spinner dolphin (*Stenella longirostris*) | 3-7/7 (43-100 %)* | [26] |
| Pygmy killer whale (*Feresa attenuata*) | 1/3 (33 %) | [26] |
| Pygmy sperm whale (*Kogia breviceps*) | 2-6/22 (9-27 %)* | [26] |
| Dwarf sperm whale (*Kogia sima)* | 0-1/6 (0-17 %)* | [26] |
| Beluga whale (*Delphinapterus leucas*) | 28/488 (6 %) | [3] |
|  | 10/147 (7 %) | [35] |
| Pilot whale (*Globicephala melas*) | 1/1 (100 %) | [8] |
| Grey whale (*Eschrichtius robustus*) | 1/1 (100 %) | [24] |
| Narwhal *(Monodon monoceros)* | 5/77 (7 %) | [3] |
| Minke whale (*Balaenoptera acutorostrata*) | 15/40 (38%)*** | [37] |
|  | 17/216 (8 %) | [2] |
| Fin whale (*Balaenoptera physalus*) | 12/108 (11 %) | [2] |
| Sei whale (*Balaenoptera borealis*) | 7/49 (14 %) | [2] |
| Killer whale (*Orcinus orca*) | 4/5 (80 %) | [24] |
|  | 1/1 (100 %) | [8] |
| Southern sea otter (*Enhydra lutris nereis*) | 4/68 (6 %) | [38] |
| Alaskan sea otter (*Enhydra lutris kenyoni*) | 5/65 (8 %) and 1/37 (3 %) | [38] |
| Polar Bear (*Ursus maritimus*) | 25/500 (5 %) | [39] |
|  | 16/297 (5 %) | [40] |
|  | 28/275 (10 %) | [41] |

* Several different serological tests used giving different results.

** Unable to find the number of animals / group in the text

*** Some animals had granular lesions in testes / uterus

Reference List

1. Tryland M, Sorensen KK, Godfroid J: **Prevalence of *Brucella* *pinnipediae* in healthy hooded seals (*Cystophora cristata*) from the North Atlantic Ocean and ringed seals (*Phoca hispida*) from Svalbard.** *Vet Microbiol* 2005, **105:**103-111.

2. Tryland M, Kleivane L, Alfredsson A, Kjeld M, Arnason A, Stuen S, Godfroid J: **Evidence of *Brucella* infection in marine mammals in the North Atlantic Ocean.** *Vet Rec* 1999, **144:**588-592.

3. Nielsen O, Stewart REA, Nielsen K, Measures L, Duignan P: **Serologic survey of *Brucella* spp. antibodies in some marine mammals of North America.** *J Wildl Dis* 2001, **37:**89-100.

4. Nielsen O, Nielsen K, Stewart REA: **Serologic evidence of *Brucella* spp. exposure in Atlantic walruses (*Odobenus rosmarus rosmarus*) and ringed seals (*Phoca hispida*) of Arctic Canada.** *Arctic* 1996, **49:**383-386.

5. Maratea J, Ewalt DR, Frasca S, Dunn JL, De Guise S, Szkudlarek L, St Aubin DJ, French RA: **Evidence of *Brucella* sp. infection in marine mammals stranded along the coast of southern New England.** *J Zoo Wildl Med* 2003, **34:**256-261.

6. Ross HM, Jahans KL, MacMillan AP, Reid RJ, Thompson PM, Foster G: ***Brucella* species infection in North Sea Seal and cetacean populations.** *Vet Rec* 1996, **138:**647-648.

7. Ross HM, Foster G, Reid RJ, Jahans KL, MacMillan AP: ***Brucella* Species Infection in Sea-Mammals.** *Vet Rec* 1994, **134:**359.

8. Jepson PD, Brew S, MacMillan AP, Baker JR, Barnett J, Kirkwood JK, Kuiken T, Robinson IR, Simpson VR: **Antibodies to *Brucella* in marine mammals around the coast of England and Wales.** *Vet Rec* 1997, **141:**513-515.

9. Garner MM, Lambourn DM, Jeffries SJ, Hall PB, Rhyan JC, Ewalt DR, Polzin LM, Cheville NF: **Evidence of *Brucella* infection in Parafilaroides lungworms in a Pacific harbor seal (*Phoca vitulina richardsi*).** *J Vet Diagn Invest* 1997, **9:**298-303.

10. Zarnke RL, Saliki JT, MacMillan AP, Brew SD, Dawson CE, Hoef JMV, Frost KJ, Small RJ: **Serologic survey for *Brucella* spp., phocid herpesvirus-1, phocid herpesvirus-2, and phocine distemper virus in harbor seals from Alaska, 1976-1999.** *J Wildl Dis* 2006, **42:**290-300.

11. Gaydos JK, Lambourn DM, Lockwood S, Jeffries SJ, Ewalt DR, Garner GW, Sidor I, Raverty SA: **Endemic Marine *Brucella* sp. Infection in Harbor Seals: Is there Human Risk?** In *45th annual meeting of Infectious Diseases Society of America: 4-7 October 2007; San Diego, USA.*

12. Lambourn DM, Jeffries SJ, Garner MM, Ewalt DR, Raverty SA, Gaydos JK, Rhyan JC: **Evidence of *Brucella* Sp. Infection in Pacific Harbor Seals (*Phoca vitulina richardsii*) 1993-2004 From Washington .** In *Pugent Sound Research Conference: 12-14 February 2001; Bellevue, USA*.

13. Burek KA, Gulland FMD, Sheffield G, Beckmen KB, Keyes E, Spraker TR, Smith AW, Skilling DE, Evermann JF, Stott JL et al.: **Infectious disease and the decline of Steller sea lions (Eumetopias jubatus) in Alaska, USA: Insights from serologic data.** *J Wildl Dis* 2005, **41:**512-524.

14. Nielsen O, Nielsen K, Braun R, Kelly L: **A comparison of four serologic assays in screening for *Brucella* exposure in Hawaiian monk seals.** *J Wildl Dis* 2005, **41:**126-133.

15. Aguirre AA, Keefe TJ, Reif JS, Kashinsky L, Yochem PK, Saliki JT, Stott JL, Goldstein T, Dubey JP, Braun R et al.: **Infectious disease monitoring of the endangered Hawaiian monk seal.** *J Wildl Dis* 2007, **43:**229-241.

16. Blank O, Retamal P, Abalos P, Torres D: **Detection of anti-*brucella* antibodies in Weddell seals (*Leptonychotes weddellii*) from cape Shirref, Antarctica.** *Archivos de Medicina Veterinaria* 2002, **34:**117-122.

17. Retamal P, Blank O, Abalos P, Torres D: **Detection of anti-*Brucella* antibodies in pinnipeds from the Antarctic territory.** *Vet Rec* 2000, **146:**166-167.

18. Abalos P, Retamal P, Blank O, Torres D, Valdenegro V: ***Brucella* infection in marine mammals in Antarctica.** *Vet Rec* 2009, **164:**250.

19. Blank O, Retamal P, Abalos P, Torres D: **Additional data on anti-*Brucella* antibodies in *Arctocephalus gazella* from Cape Shirreff, Livingston Island, Antarctica.** *CCAMLR Science* 2001, **8:**147-154.

20. Lynch M, Duignan PJ, Taylor T, Nielsen O, Kirkwood R, Gibbens J, Arnould JPY: **Epizootiology of *Brucella* infection in Australian fur seals.** *J Wildl Dis* 2011, **47:**352-363.

21. Dawson CE: **Anti-*Brucella* antibodies in pinnipeds of Australia.** *Microbiology Austraila* 2005, **26:**87-89.

22. Neimanis AS, Koopman HN, Westgate AJ, Nielsen K, Leighton FA: **Evidence of exposure to *Brucella* sp in harbor porpoises (*Phocoena phocoena*) from the Bay of Fundy, Canada.** *J Wildl Dis* 2008, **44:**480-485.

23. Dawson CE, Perrett LL, Stubberfield EJ, Stack JA, Farrelly SSJ, Cooley WA, Davison NJ, Quinney S: **Isolation and characterization of *Brucella* from the lungworms of a harbor porpoise (*Phocoena phocoena*).** *J Wildl Dis* 2008, **44:**237-246.

24. Raverty SA, Gaydos JK, Nielsen K, Nielsen O, Ross PS, Lambourn DM, Jeffries SJ: **An Overview of Marine Mammal Diagnoses in the Pacific Northwest from 1999 to 2004 .** In *World Small Animal Veterinary Association World Congress: 8-11 August 2002; Vancouver, Canada.*

25. Van Bressem MF, Van Waerebeek K, Raga JA, Godfroid J, Brew SD, MacMillan AP: **Serological evidence of *Brucella* species infection in odontocetes from the south Pacific and the Mediterranean.** *Vet Rec* 2001, **148:**657-661.

26. Hernandez-Mora G, Manire CA, Gonzalez-Barrientos R, Barquero-Calvo E, Guzman-Verri C, Staggs L, Thompson R, Chaves-Olarte E, Moreno E: **Serological Diagnosis of *Brucella* Infections in Odontocetes.** *Clin Vaccine Immunol* 2009, **16:**906-915.

27. Hernandez-Mora G, Gonzalez-Barrientos R, Morales JA, Chaves-Olarte E, Guzman-Verri C, Baquero-Calvo E, De-Miguel MJ, Marin CM, Blasco JM, Moreno E: **Neurobrucellosis in Stranded Dolphins, Costa Rica.** *Emerg Infect Dis* 2008, **14:**1825.

28. Gonzalez L, Patterson IA, Reid RJ, Foster G, Barberan M, Blasco JM, Kennedy S, Howie FE, Godroid J, MacMillan AP et al.: **Chronic meningoencephalitis associated with *Brucella* sp. infection in live-stranded striped dolphins (*Stenella coeruleoalba*).** *J Comp Pathol* 2002, **126:**147-152.

29. Gonzalez-Barrientos R, Morales JA, Hernandez-Mora G, Barquero-Calvo E, Guzman-Verri C, Chaves-Olarte E, Moreno E: **Pathology of Striped Dolphins (*Stenella coeruleoalba*) Infected with *Brucella ceti*.** *J Comp Pathol* 2010, **142:**347-352.

30. Davison NJ, Cranwell MP, Perrett LL, Dawson CE, Deaville R, Stubberfield EJ, Jarvis DS, Jepson PD: **Meningoencephalitis associated with *Brucella* species in a live-stranded striped dolphin (*Stenella coeruleoalba*) in south-west England.** *Vet Rec* 2009, **165:**86-89.

31. Munoz PM, Garcia-Castrillo G, Lopez-Garcia P, Gonzalez-Cueli JC, De Miguel MJ, Marin CM, Barberan M, Blasco JM: **Isolation of *Brucella* species from a live-stranded striped dolphin (*Stenella coeruleoalba*) in Spain.** *Vet Rec* 2006, **158:**450-451.

32. Dawson CE, Perrett LL, Young EJ, Davison NJ, Monies RJ: **Isolation of *Brucella* species from a bottlenosed dolphin (*Tursiops truncatus*).** *Vet Rec* 2006, **158:**831-832.

33. Miller WG, Adams LG, Ficht TA, Cheville NF, Payeur JP, Harley DR, House C, Ridgway SH: ***Brucella*-induced abortions and infection in bottlenose dolphins (*Tursiops truncatus*).** *J Zoo Wildl Med* 1999, **30:**100-110.

34. Alekseev AY, Rozanova EI, Ustinova EN, Tumanov YI, Kuvshinova IN, Shestopalov AM: **The prevalence of antibodies to morbilliviruses, *Brucella*, and Toxoplasma in the Black Sea bottlenose dolphin *Tursiops truncatus ponticus* maintained in captivity.** *Russian Journal of Marine Biology* 2007, **33:**425-428.

35. Alekseev AY, Reguzova AY, Rozanova EI, Abramov AV, Tumanov YV, Kuvshinova IN, Shestopalov AM: **Detection of specific antibodies to morbilliviruses, *Brucella* and Toxoplasma in the Black Sea dolphin *Tursiops truncatus ponticus* and the beluga whale *Delphinapterus leucas* from the Sea of Okhotsk in 2002-2007.** *Russian Journal of Marine Biology* 2009, **35:**494-497.

36. Tachibana M, Watanabe K, Kim S, Omata Y, Murata K, Hammond T, Watarai M: **Antibodies to *Brucella* spp. in Pacific bottlenose dolphins from the Solomon Islands.** *J Wildl Dis* 2006, **42:**412-414.

37. Ohishi K, Zenitani R, Bando T, Goto Y, Uchida K, Maruyama T, Yamamoto S, Miyazaki N, Fujise Y: **Pathological and serological evidence of *Brucella*-infection in baleen whales (Mysticeti) in the western North Pacific.** *Comp Immunol Microbiol Infect Dis* 2003, **26:**125-136.

38. Hanni KD, Mazet JAK, Gulland FMD, Estes J, Staedler M, Murray MJ, Miller M, Jessup DA: **Clinical pathology and assessment of pathogen exposure in southern and Alaskan sea otters.** *J Wildl Dis* 2003, **39:**837-850.

39. Rah H, Chomel BB, Follmann EH, Kasten RW, Hew CH, Farver TB, Garner GW, Amstrup SC: **Serosurvey of selected zoonotic agents in polar bears (*Ursus maritimus*).** *Vet Rec* 2005, **156:**7-13.

40. Tryland M, Derocher AE, Wiig O, Godfroid J: ***Brucella* sp. antibodies in polar bears from Svalbard and the Barents Sea.** *J Wildl Dis* 2001, **37:**523-531.

41. O´Hara TM, Holcomb D, Elzer P, Estepp J, Perry Q, Hagius S, Kirk C: ***Brucella* species survey in polar bears (Ursus maritimus) of Northern Alaska**. *J Wildl Dis* 2010, **46:** 687-694
